# The Role of Matrix Metalloproteinase Single-Nucleotide Polymorphisms in the Clinicopathological Properties of Breast Cancer

**DOI:** 10.3390/biomedicines10081891

**Published:** 2022-08-04

**Authors:** Agnė Bartnykaitė, Aistė Savukaitytė, Justina Bekampytė, Rasa Ugenskienė, Danguolė Laukaitienė, Erika Korobeinikova, Jurgita Gudaitienė, Elona Juozaitytė

**Affiliations:** 1 Oncology Research Laboratory, Oncology Institute, Lithuanian University of Health Sciences, LT-50161 Kaunas, Lithuania; 2 Department of Genetics and Molecular Medicine, Lithuanian University of Health Sciences, LT-50161 Kaunas, Lithuania; 3 Oncology Institute, Lithuanian University of Health Sciences, LT-50161 Kaunas, Lithuania

**Keywords:** breast cancer, SNP, MMP, association

## Abstract

(1) Background. Breast cancer is the leading cancer type among women. Despite convenient diagnostics at early stages, there is a need for continuous monitoring to predict more aggressive or recurring breast cancer forms. The evidence suggests that the detection of genetic biomarkers could help in improving disease management and reduce mortality. Matrix metalloproteinases (MMPs) are a large family of enzymes that perform physiologically relevant functions and have the potential properties to be biomarkers for cancer assessment. We aimed to evaluate the contribution and association of single-nucleotide polymorphisms (SNPs) in *MMP* genes (*MMP1*, *MMP2*, *MMP3*, *MMP7*, *MMP8*, *MMP9*) with clinicopathological breast-cancer features. (2) Methods. In this study, 100 breast cancer patients were genotyped by polymerase chain reaction–restriction fragment length polymorphism methodology (PCR–RFLP). (3) Results. The presence of the *MMP7* rs11568818 A allele was associated with lower chances for poorly differentiated breast cancer. The lower possibility for HER2-positive breast cancer was associated with the presence of the *MMP9* rs3918242 C allele. (4) Conclusions. These results indicate that *MMP7* rs11568818 and *MMP9* rs3918242 are potential biomarkers for the anticipation of breast cancer aggressiveness.

## 1. Introduction

Breast cancer (BC) is globally the most common cancer among women, making this disease a leading cause of morbidity and mortality [1]. Despite many tools in early diagnostics, therapy, and prevention, BC is a very heterogeneous disease that poses challenges to treatment and the prediction of outcomes [2,3]. Tumors of BC differ in their clinical and histopathological features, biomarkers, and genetic profiles. Biomarkers that enable the distinction of a more aggressive form of BC would help in the determination of patients’ prognosis and in choosing a more appropriate treatment [3,4,5,6].

Matrix metalloproteinases (MMPs) are a family of proteolytic enzymes that play a crucial role in many physiological processes owing to their tremendous capacity to degrade extracellular proteins. At least 23 of these multidomain zinc-dependent endopeptidases are expressed in humans [7,8,9,10]. Imbalances in the MMP system regarding different gene variants and altered gene expression levels are linked to several pathologies, one of which is cancer. One of the best studied MMPs is MMP1. Despite important physiological functions, clinical studies found associations between changes in MMP1 and various cancers [11,12,13]. One of the studied single-nucleotide polymorphisms (SNPs) in the *MMP1* promoter region is rs1799750, which controls the transcriptional activity of *MMP1,* and was associated with the incidence and progression of several types of cancer [14,15,16,17,18,19,20]. Several polymorphisms have been studied in the *MMP2* gene. The results of SNPs and expression analysis showed associations with cancers, including BC [21,22]. rs3025058, located in *MMP3*, is also regarded as one of the potential genetic factors for BC assessment in different populations [14,23,24]. The studies showed that the overexpression of MMP7 is common for a wide variety of cancers. Moreover, SNPs in *MMP7* are associated with an increased risk of cancer development, tumor characteristics and the course of disease [25,26]. It is hypothesized that different variants in the promoter region of *MMP8* may also influence cancer risk and prognosis [27,28,29]. Furthermore, associations between polymorphisms and clinical features were found in BC patients assessing the effect of *MMP9* [14,21,24,29,30]. Taken together, the role of MMPs in cancer development renders them a prominent target. Therefore, we investigated the correlation between several polymorphisms in *MMPs* and BC clinicopathological features. The results could provide new information, and the studied SNPs might be the useful genetic markers for patient stratification in the future.

## 2. Materials and Methods

### 2.1. Study Population

This association study comprised 100 premenopausal 30–50-year-old women diagnosed with stage I–II BC. All subjects were of Lithuanian nationality and were recruited for this study from the Hospital of Lithuanian University of Health Sciences Kauno klinikos, Kaunas, Lithuania. The exclusion criteria were other malignancies, significant comorbidities, and incomplete medical documentation. The participants received and signed a consent form before entering the study. The research was approved by the Kaunas Regional Biomedical Research Ethics Committee (2014-05-07 protocol no. BE-2-10 and 2015-12-31 protocol no. P1-BE-2-10/2014).

### 2.2. DNA Extraction and Polymerase Chain Reaction—Restriction Fragment Length Polymorphism (PCR-RFLP)

Blood samples were collected in EDTA-containing tubes from all included subjects. Genomic DNA was extracted from peripheral blood leukocytes using a commercially available DNA extraction kit (ThermoFisher Scientific Baltics, Vilnius, Lithuania) according to the manufacturer’s instructions. Isolated DNA was stored at −20 °C until analysis.

SNPs were analyzed with the polymerase chain reaction–restriction fragment length polymorphism (PCR–RFLP) method. PCR was performed in a total volume of 25 µL. Conditions for each SNP of interest were optimized individually. The PCR profile for *MMP1* rs1799750 polymorphism was 7 min of denaturation at 95 °C, then 35 cycles of 95 °C for 30 s, 51 °C for 1 min, and 72 °C for 35 s. The final cycle had a 7 min extension at 72 °C. For *MMP2* rs243865 analysis, PCR cycling conditions were 94 °C for 5 min following 40 cycles (94 °C 30 s, 60.9 °C 30 s, 72 °C 30 s) amplification with the final step at 72 °C for 10 min. In order to analyze *MMP3* rs3025058 and *MMP9* rs3918242 polymorphisms, DNA fragments were amplified by PCR using the following conditions: 95 °C for 5 min following 35 cycles (95 °C 30 s, 66 °C 30 s, 72 °C 30 s) of polymerization and finishing with a final extension at 72 °C for 5 min. For *MMP7* rs11568818 polymorphism determination, the thermal cycling program was employed as follows: 95 °C for 7 min, followed by 35 cycles (95 °C 30 s, 49 °C 1 min, and 72 °C 35 s), and a final elongation step at 72 °C for 7 min. For *MMP8* rs11225395 polymorphism detection, PCR conditions were 94 °C for 5 min followed by 38 cycles (94 °C 30 s, 56.6 °C 30 s, 72 °C 30 s) with a final step at 72 °C for 10 min. The amplification reactions were followed by visualization on 3% agarose gel in order to ensure the size of the product. PCR products were digested at 37 °C, subjected to 3% agarose gel electrophoresis, and visualized by ethidium bromide staining. PCR primer sequences, digestion enzymes for each SNP, and sizes of digested DNA fragments are depicted in Table 1. 

### 2.3. Statistical Analysis

The deviation from Hardy–Weinberg equilibrium (HWE) was tested. Pearson’s chi-squared or Fisher’s exact tests were used to determine the relationships between genotypes and clinicopathological characteristics. Differences in allele and genotype distributions were analyzed using logistic regression. The odds ratio (OR) and 95% confidence interval (CI) were used as a measure of the strength of association between polymorphism and characteristic. SNP association analyses were conducted using univariate logistic regression, followed by multivariate regression for significant positive associations. The multivariate analyses were performed in three models (1–3). Model 1 included SNP and age at diagnosis as a potential covariate. In Model 2, the receptors as additional confounding variables were involved. In Model 3, tumor size and lymph node involvement as additional confounding variables were included. When the *p*-value was <0.05, results were considered to be statistically significant. Survival curves were estimated using the Kaplan–Meier method. Statistical significance was assessed using the log-rank test. Statistical analysis was carried out using IBM SPSS Statistics 27.0.1 statistical software.

## 3. Results

### 3.1. Cohort Characteristics and Genotypes Distribution

A total of 100 BC patients were included in this study with the prior consent of each individual. The group consisted only of women. The average age of the subjects was 42.21 ± 5.51 years, and the age range was 30–50 years. In this study, we searched for associations among six genetic variants of *MMPs* (*MMP1* rs1799750, *MMP2* rs243865, *MMP3* rs3025058, *MMP7* rs11568818, *MMP8* rs11225395, *MMP9* rs3918242) and characteristics including patient age at diagnosis, tumor size (T), the status of lymph node involvement (N), the status of estrogen (ER), progesterone (PR), and human epidermal growth factor receptor 2 (HER2) receptors, tumor histological type (ductal, lobular, medullary, and other) and grade (G1–G2 and G3), progression, metastasis, and death (Table 2). The frequencies of the relevant distribution of genotype variants in the studied population were similar to published data of the European Population Allele Frequencies from the 1000 Genomes Project Database and are listed in Table 3. SNP genotype frequencies were in HWE.

### 3.2. Associations of SNPs with BC

We aimed to determine the link between various SNPs in *MMP* genes and clinicopathological characteristics of BC, and disease progression, metastasis, and death. None of the studied SNPs showed any significant association with age at breast cancer diagnosis, tumor size (T), lymph node involvement (N), the status of estrogen receptor (ER), tumor histological type, progression, metastasis, and death. However, *MMP1* rs1799750 and *MMP7* rs11568818 were significantly associated with tumor histological grade (*p* < 0.05). Meanwhile, *MMP8* rs11225395 was associated with the status of progesterone receptor (PR), while *MMP9* rs3918242 was significantly associated with the status of HER2 receptor (*p* < 0.05) (Supplementary Materials Table S1). 

Univariate logistic regression analysis showed that patients with *MMP1* rs1799750 1G1G (versus 2G2G) genotype and 2G allele noncarriers (versus carriers) were predisposed to lower rates of poorly differentiated BC (OR = 0.095, 95% CI 0.022–0.406, *p* = 0.001, and OR = 0.176, 95% CI 0.038–0.810, *p* = 0.026, respectively). Moreover, univariate analysis revealed that GG genotype of *MMP7* rs11568818 showed a strong association (OR = 6.562, 95% CI 1.532–28.120, *p* = 0.011) with higher chances for poor differentiation of BC, compared with AA genotype. Similar results were determined analyzing rs11568818 A allele noncarriers versus carriers (OR = 5.330, 95% CI 1.424–19.941, *p* = 0.013) (Table 4). 

The association between *MMP1* rs1799750 and G3 histological grade remained statistically significant in a multivariate logistic regression analysis Model 1, where age at diagnosis was included as a potential confounding variable. The results were very similar to those obtained from univariate logistic regression analysis. However, further adjustments for tumor receptors status (Model 2) and other variables (Model 3) resulted in the loss of significance (Table 5). Meanwhile, *MMP7* rs11568818 was statistically significantly associated with poorly differentiated breast cancer in all three analyzed multivariate logistic regression models. Patients carrying the GG genotype compared to AA were predisposed to higher rates of G3 histological grade cancer: following adjustment for age at diagnosis (OR = 6.593, 95% CI 1.536–28.304, *p* = 0.011); age and status of tumor receptors (OR = 7.099, 95% CI 1.382–36.456, *p* = 0.019); age, status of tumor receptors, tumor size and lymph node involvement (OR = 8.425, 95% CI 1.286–55.200, *p* = 0.026). In addition, in spite of the other tumor characteristics, the poor cancer differentiation was more frequently presented in A allele noncarriers than in carriers: in multivariate analysis Model 1, OR = 5.330, 95% CI 1.423–19.968, *p* = 0.013, Model 2, OR = 6.568, 95% CI 1.471–29.320, *p* = 0.014, Model 3, OR = 9.307, 95% CI 1.568–55.253, *p* = 0.014.

*MMP8* rs11225395 did not show statistically significant association with negative status of progesterone receptor in the univariate logistic regression analysis (*p* > 0.05). Meanwhile, results show that the presence of *MMP9* rs3918242 C allele was significantly associated with decreased chances of HER2 receptor-positive BC (OR = 0.256, 95% CI 0.157–0.419, *p* = 0.000) (Table 4). As indicated in Table 5, this association remained statistically significant in the multivariate analysis: Model 1, OR = 0.200, 95% CI 0.104–0.382, *p* = 0.000, Model 2, OR = 0.143, 95% CI 0.061–0.336, *p* = 0.000, Model 3, OR = 0.104, 95% CI 0.036–0.301, *p* = 0.000.

**Figure 1:**
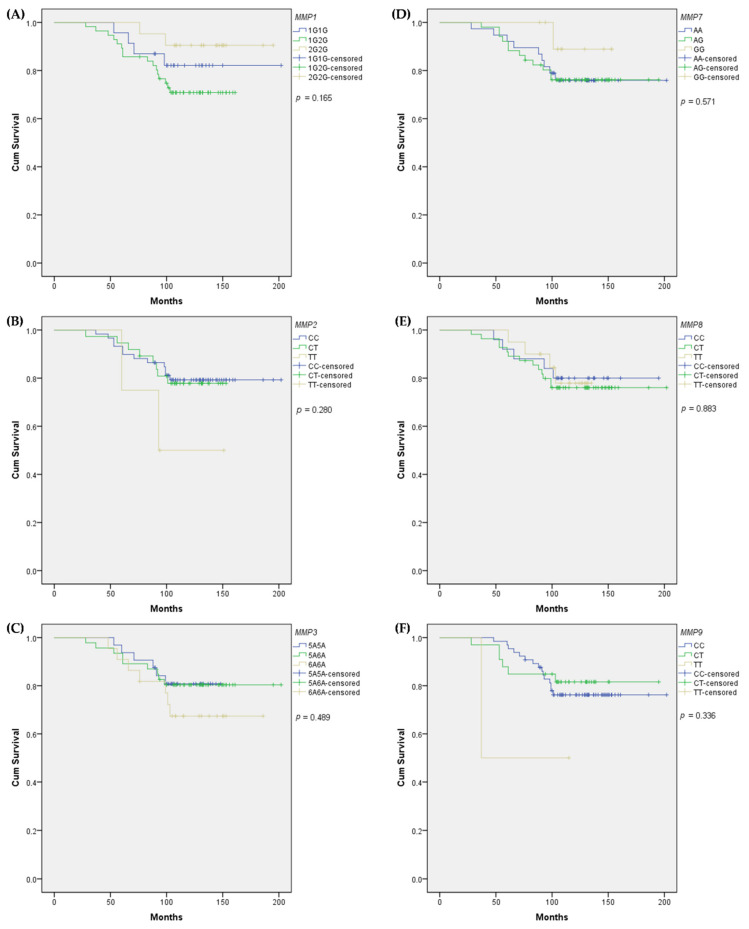
Kaplan–Meier estimates of overall survival: (**A**) *MMP1* rs1799750, (**B**) *MMP2* rs243865, (**C**) *MMP3* rs3025058, (**D**) *MMP7* rs11568818, (**E**) *MMP8* rs11225395, (**F**) *MMP9* rs3918242.

To assess the prognostic role of selected SNPs, we further tested their association with overall survival. However, no significant association was detected between all analyzed SNPs and patients’ overall survival (log-rank, *p* > 0.05) (Figure 1).

## 4. Discussion

Breast cancer (BC) is the leading cause of cancer-related women’s death. MMP upregulation is associated with poor outcomes in BC [21,22,37]. Changes in gene expression may be influenced by polymorphisms in the promoter region. Additionally, single-nucleotide polymorphisms in *MMP* genes have been reported for their possible association with various cancers. The determined relation of SNPs to specific disease characteristics might significantly impact applying a more suitable treatment and improving cancer prognosis [26,38]. The purpose of the present study was to evaluate the correlation of SNPs in *MMP* genes to well-known clinicopathological features and course of disease in patients with breast cancer.

Although *MMP1* is one of the best-studied MMPs, there are not enough studies to develop a strong attitude concerning associations between SNPs and clinical breast cancer features. In the current study, our findings from univariate analysis showed that *MMP1* rs1799750 2G allele noncarriers were significantly less linked to poor breast cancer differentiation. However, the adjusted odds ratios resulted in the loss of significance, suggesting that other factors were more important in this case. Other results are in accordance with earlier studies carried out on breast cancer. Zhou et al. [39], Padala et al. [14], Przybylowska et al. [12], Lei et al. [30], and Ghilardi et al. [23] did not show any associations between the genotype distribution of *MMP1* rs1799750 and age, tumor size, tumor stage and type, ER/PR status, lymph node status, distant metastasis, ethnicity, and menopausal status. However, in contrast to our results, they did not notice the association with histological grade. Meanwhile, Zhou et al. [39] claimed that the 2G2G genotype, compared to 1G2G, was associated with HER2 expression (OR = 7.5, 95% CI 1.48–37.9, *p* = 0.032). On the other hand, the results obtained from the study of Padala with colleagues [14] showed that the 2G2G genotype tends to be associated with decreased chances of HER2 receptor-positive breast cancer (OR = 0.43, 95% CI 0.18–1.04, *p* = 0.05). Hughes and colleagues [15] demonstrated that BC patients carrying the 2G2G genotype had a significant, almost threefold, increased risk of lymph node metastasis compared with subjects carrying the 1G1G genotype (OR = 2.9, 95% CI 1.4–6.2, *p* = 0.01). Similar results were obtained by Przybylowska et al. [12]. Their study showed that the 2G2G genotype and 2G allele were related to the higher risk of metastasis development in lymph node (OR = 2.14, 95% CI 1.24–3.69, *p* < 0.05 and OR = 1.68, 95% CI 1.19–2.39, *p* < 0.05, respectively).

In our study, *MMP2* rs243865 did not show any statistically significant associations with the analyzed BC features. Our findings agree with studies of Habel et al. [40], and Saeed et al. [17], which did not reveal associations between rs243865 and status of ER, PR and HER2, tumor size, distant metastasis, nodal status, molecular type, and menarche. However, in Habel et al. [40], rs243865 was positively associated with menstrual irregularity (*p* = 0.005) and histological type (*p* = 0.002). Meanwhile, Saeed et al. [17] found that rs243865 CC was linked to the age of older patients (above 48 years) (OR = 2.35, 95% CI 1.02–5.39, *p* = 0.038). 

The other SNP involved in this study, *MMP3* rs3025058, showed no associations with the analyzed BC characteristics. In concordance with our findings, Padala et al. [14], AbdRaboh et al. [24], Lei et al. [30], and Krippl et al. [41] did not observe any associations with type of cancer, age at diagnosis, tumor size, the status of ER, PR and HER2/neu, histological grade, the status of distant metastasis. However, there are some conflicting results regarding associations with cancer stage and status of lymph nodes. A study by AbdRaboh et al. [24] showed that *MMP3* rs3025058 6A allele carriers had an almost threefold increased risk for advanced (III–IV)-stage cancer (OR = 2.9, 95% CI 1.04–8.45, *p* < 0.05) but, in concordance to our findings, did not reveal association with the status of lymph nodes. Meanwhile, Padala et al. [14] demonstrated no association with the stage of cancer, but in their study, the rs3025058 5A6A (versus 5A5A) genotype was significantly associated with lymph-node-positive cases (OR = 2.58, 95% CI 1.39–4.80, *p* = 0.01). 

Concerning *MMP7*, rs11568818 A allele noncarriers were predisposed to considerably higher rates of G3-histological-grade cancer in all analyzed statistical models. Other findings are consistent with the study of Beeghly-Fadiel et al. [25], where this SNP was not associated with patients’ age, stage of disease, menopausal status, and >ER and PR status. Additionally, Yari with colleagues [42] did not reach statistical significance when analyzing associations between rs11568818 and lymph node metastasis, the status of ER and PR, and histological type in patients from western Iran.

The most studied SNP in the *MMP8* gene is rs11225395. In the present study, the investigated association of rs11225395 and PR status resulted in a loss of significance in logistic regression analysis. By contrast, Decock et al. [28] noted that the T allele predisposes to lower rates of lymph node metastasis (*p* = 0.02), but no associations were found between SNP and tumor size, histological grade, tumor subtype, or cancer stage. However, to our knowledge, there are no more studies evaluating the associations of rs11225395 and BC clinicopathological features. Consequently, future studies are needed to confirm or refute these results.

The analysis of *MMP9* rs3918242 indicated that it is associated with HER2 status. The lower possibility of the positive status of HER2 was related to the presence of the C allele. Padala and colleagues [14] did not notice any association between *MMP9* rs3918242 and stage or type of cancer, lymph node status, the status of ER, PR and HER2/neu, status of distant metastasis. Hughes et al. [15] demonstrated that subjects carrying the CT genotype had a significant, over threefold, increased risk of lymph node metastasis compared with patients carrying the CC genotype (OR = 3.4, 95% CI 1.3–8.9, *p* = 0.01). Moreover, a study by AbdRaboh et al. [24] demonstrated the associations of patients with *MMP9* rs3918242 CT + TT genotypes to increased risk of larger (OR = 3.2, 95% CI 1.17–8.99, *p* < 0.05) and low histological grade (OR = 2.4, 95% CI 0.82–6.84, *p* < 0.05) tumors. Moreover, in line with our findings, the authors did not report any significant associations with cancer stage, the status of lymph nodes, and metastasis.

In the overall survival study, we did not notice any significant associations between the genotypes of analyzed SNPs and survival. Our findings concur with the studies of Lei et al. [30], and Padala et al. [14], in which *MMP1* rs1799750, *MMP2* rs243865, *MMP3* rs3025058, and *MMP9* rs3918242 did not correlate with survival in a breast cancer patients’ group. However, Hughes and colleagues [15] observed that the *MMP1* rs1799750 2G2G genotype was associated with worse overall survival (HR = 3.1, 95% CI 1.1–8.7, *p* = 0.03). In the study of Beeghly-Fadiel et al. [25], *MMP7* rs11568818 GG genotype carriers tended to have worse disease-free (HR = 5.5, 95% CI 2.1–14.8, *p* < 0.05) and overall survival (HR = 5.8, 95% CI 2.1–15.0, *p* < 0.05). Moreover, *MMP8* rs11225395 CT + TT was associated with reduced breast cancer relapse (*p* = 0.04), greater overall HR = 0.7, 95% CI 0.5–1.0, *p* = 0.02) and disease-free survival (HR = 0.7, 95% CI 0.5–0.9, *p* = 0.02) among women with stage 0–II cancer in the Shanghai Breast Cancer Study [28].

## 5. Conclusions

Our findings showed that *MMP7* rs11568818 A allele carriers were associated with better prognosis regarding lower chances for poorly differentiated breast cancer. Moreover, a better prognosis concerning lower chances for positive HER2 status was associated with the presence of the *MMP9* rs3918242 C allele. Although there are several studies that investigated the associations between SNPs in *MMP* genes and clinicopathological features of breast cancer, the results are not fully sufficient to reach a consensus. Further studies with a larger sample and functional research should be conducted to confirm our findings and better elucidate the underlying biological mechanisms. 

## Figures and Tables

**Table 1:** Primer sequences for analyzed gene polymorphisms, length of PCR–RFLP products, and restriction enzymes.

Polymorphism	Primer Sequence *	Length of Fragments, Bp		Restriction Enzymes	Ref.
*MMP1* rs1799750	F: 5′-TGACTTTTAAAACATAGTCTATGTTCA-3′ R: 5′-TCTTGGATTGATTTGAGATAAGTCATAGC-3′	2G allele 270	1G allele 241 + 28	FastDigest AluI	[31]
*MMP2* rs243865	F: 5′-ATATTCCCCACCCAGCAGTC-3′ R: 5′-TTGGGAACGCCTGACTTCAG-3′	C allele 122	T allele 103 + 19	AccI	[32]
*MMP3* rs3025058	F: 5′-GGTTCTCCATTCCTTTGATGGGGGGAAAGA-3′ R: 5′-CTTCCTGGAATTCACATCACTGCCACCACT-3′	5A allele 96 + 33	6A allele 130	FastDigest PsyI	[31,33]
*MMP7* rs11568818	F: 5′-TGGTACCATAATGTCCTGAATG-3′ R: 5′-TCGTTATTGGCAGGCCGCACACAATGAATT-3′	A allele 150	G allele 130 + 20	EcoRI	[34]
*MMP8* rs11225395	F: 5′-CCATCTTCACATAGCCTTGG-3′ R: 5′-CCTTGTCTTCTGCCTGTGAA-3′	C allele 172 + 113	T allele 285	BfmI	[35]
*MMP9* rs3918242	F: 5′-GCCTGGCACATAGTAGGCCC-3′ R: 5′-CTTCCTAGCCAGCCGGCATC-3′	C allele 436	T allele 242 + 194	SphI	[36]

F—forward primer, R—reverse primer, Ref.—references, * nucleotide base in the underlined letter is mismatched base added in the primer sequence to create restriction enzyme site.

**Table 2:** Characteristics of the study group (*n* = 100).

Characteristic	Subgroups and Frequencies
Patient age at diagnosis	30–40 years—35%, 40–50 years—65%
Tumor size (T)	0–2 cm—66%, >2–5 cm—34%
Lymph node involvement (N)	negative—54%, positive—46%
Estrogen receptor (ER)	negative—43%, positive—57%
Progesterone receptor (PR)	negative—52%, positive—48%
Human epidermal growth factor receptor 2 (HER2)	negative—78%, positive—22%
Tumor histological type	ductal—88%, lobular—4%, medullary—3%, other—5%
Tumor histological grade (G)	G1 or G2—71%, G3—29%
Progression	absent—69%, present—31%
Metastasis	absent—74%, present—26%
Death	absent—78%, present—22%

G1—well differentiated (low grade), G2—moderately differentiated (intermediate grade), G3—poorly differentiated (high grade).

**Table 3:** Genotypes distribution of *MMPs* polymorphisms in the study group (*n* = 100).

SNP	Genotype Frequency		
*MMP1* rs1799750	2G2G 0.21	1G2G 0.56	1G1G 0.23
*MMP2* rs243865	CC 0.59	CT 0.37	TT 0.04
*MMP3* rs3025058	5A5A 0.32	5A6A 0.46	6A6A 0.22
*MMP7* rs11568818	AA 0.38	AG 0.51	GG 0.11
*MMP8* rs11225395	CC 0.25	CT 0.55	TT 0.20
*MMP9* rs3918242	CC 0.65	CT 0.33	TT 0.02

**Table 4:** Univariate logistic regression analysis. The odds ratio for association between SNP and tumor characteristic.

Characteristic	SNP	Genotype	OR	95% CI	*p*
G3 histological grade	*MMP1* rs1799750	2G2G	1.000	(ref.)	-
		1G2G	0.600	0.349–1.031	0.064
		1G1G	0.095	0.022–0.406	0.001
		1G1G	1.000	(ref.)	-
		1G2G + 2G2G	5.670	1.235–26.030	0.026
		1G2G + 2G2G	1.000	(ref.)	-
		1G1G	0.176	0.038–0.810	0.026
G3 histological grade	*MMP7* rs11568818	AA	1.000	(ref.)	-
		AG	1.419	0.526–3.831	0.490
		GG	6.562	1.532–28.120	0.011
		GG	1.000	(ref.)	-
		AA + AG	0.328	0.203–0.532	0.000
		AA + AG	1.000	(ref.)	-
		GG	5.330	1.424–19.941	0.013
Negative PR	*MMP8* rs11225395	TT	1.000	(ref.)	-
		CC + CT	1.353	0.868–2.108	0.181
		CC + CT	1.000	(ref.)	-
		TT	0.429	0.165–1.115	0.082
Positive HER2	*MMP9* rs3918242	CC	1.000	(ref.)	-
		CT	0.422	0.129–1.386	0.155
		TT	4.947 × 10^9^	0.000-inf	NA
		TT	1.000	(ref.)	-
		CC + CT	0.256	0.157–0.419	0.000
		CC + CT	1.000	(ref.)	-
		TT	6.300 × 10^9^	0.000-inf	NA

OR—odds ratio, CI—confidential interval, ref.—reference, inf—infinity, NA—not available, G3—poorly differentiated cancer, PR—progesterone receptors, HER2—human epidermal growth factor receptor 2.

**Table 5:** Multivariate logistic regression analysis for significant positive associations assessed in the univariate analysis.

Dependent	Covariates		Model 1			Model 2			Model 3		
			OR	95% CI	*p*	OR	95% CI	*p*	OR	95% CI	*p*
G3 histological grade	*MMP1*	1G2G vs. 2G2G	0.617	0.320–1.191	0.150	1.636	0.488–5.486	0.425	1.171	0.323–4.245	0.811
		1G1G vs. 2G2G	0.098	0.022–0.430	0.002	0.381	0.060–2.422	0.307	0.295	0.042–2.098	0.223
	Age (30–40 vs. 41–50 years)		0.932	0.373–2.332	0.881	0.784	0.273–2.252	0.651	0.724	0.233–2.249	0.577
	ER (− vs. +)					1.029	0.329–3.223	0.961	1.517	0.458–5.022	0.495
	PR (− vs. +)					5.725	1.585–20.685	0.008	4.937	1.324–18.408	0.017
	HER2 (+ vs. −)					0.411	0.111–1.521	0.183	0.312	0.077–1.261	0.102
	T (>2–5 cm vs. 0–2 cm)								2.785	0.923–8.406	0.069
	N (N1 vs. N0)								2.208	0.759–6.421	0.146
G3 histological grade	*MMP1*	1G2G + 2G2G vs. 1G1G	5.679	1.236–26.092	0.026	3.786	0.762–18.801	0.104	3.811	0.692–20.976	0.124
	Age (30–40 vs. 41–50 years)		1.207	0.478–3.048	0.690	0.856	0.306–2.396	0.767	0.746	0.247–2.254	0.603
	ER (− vs. +)					1.064	0.343–3.303	0.915	1.545	0.472–5.053	0.472
	PR (− vs. +)					5.483	1.541–19.514	0.009	4.886	1.318–18.113	0.018
	HER2 (+ vs. −)					0.429	0.117–1.569	0.201	0.314	0.078–1.266	0.103
	T (>2–5 cm vs. 0–2 cm)								2.859	0.965–8.471	0.058
	N (N1 vs. N0)								2.216	0.762–6.446	0.144
G3 histological grade	*MMP1*	1G1G vs. 1G2G + 2G2G	0.176	0.038–0.809	0.026	0.264	0.053–1.312	0.104	0.262	0.048–1.444	0.124
	Age (30–40 vs. 41–50 years)		1.207	0.478–3.048	0.690	0.856	0.306–2.396	0.767	0.746	0.247–2.254	0.603
	ER (− vs. +)					1.064	0.343–3.303	0.915	1.545	0.472–5.053	0.472
	PR (− vs. +)					5.483	1.541–19.514	0.009	4.886	1.318–18.113	0.018
	HER2 (+ vs. −)					0.429	0.117–1.569	0.201	0.314	0.078–1.266	0.103
	T (>2–5 cm vs. 0–2 cm)								2.859	0.965–8.471	0.058
	N (N1 vs. N0)								2.216	0.762–6.446	0.144
G3 histological grade	*MMP7*	AG vs. AA	1.429	0.529–3.865	0.481	1.140	0.381–3.406	0.815	0.821	0.250–2.693	0.745
		GG vs. AA	6.593	1.536–28.304	0.011	7.099	1.382–36.456	0.019	8.425	1.286–55.200	0.026
	Age (30–40 vs. 41–50 years)		1.213	0.476–3.087	0.686	0.859	0.303–2.438	0.775	0.829	0.270–2.550	0.744
	ER (− vs. +)					0.905	0.282–2.900	0.866	1.343	0.397–4.546	0.635
	PR (− vs. +)					8.069	2.084–31.249	0.003	6.587	1.697–25.563	0.006
	HER2 (+ vs. −)					0.367	0.099–1.359	0.133	0.231	0.052–1.026	0.054
	T (>2–5 cm vs. 0–2 cm)								3.019	0.945–9.649	0.062
	N (N1 vs. N0)								2.595	0.810–8.317	0.109
G3 histological grade	*MMP7*	AA + AG vs. GG	0.297	0.165–0.535	0.000	0.124	0.048–0.324	0.000	0.059	0.017–0.205	0.000
	Age (30–40 vs. 41–50 years)		1.319	0.543–3.207	0.541	0.841	0.303–2.335	0.740	0.759	0.251–2.289	0.624
	ER (− vs. +)					0.868	0.276–2.736	0.809	1.283	0.383–4.300	0.686
	PR (− vs. +)					7.907	2.103–29.731	0.002	6.117	1.598–23.413	0.008
	HER2 (+ vs. −)					0.362	0.097–1.343	0.129	0.242	0.055–1.058	0.059
	T (>2–5 cm vs. 0–2 cm)								2.833	0.892–8.998	0.077
	N (N1 vs. N0)								2.395	0.764–7.504	0.134
G3 histological grade	*MMP7*	GG vs. AA + AG	5.330	1.423–19.968	0.013	6.568	1.471–29.320	0.014	9.307	1.568–55.253	0.014
	Age (30–40 vs. 41–50 years)		1.198	0.472–5.014	0.704	0.869	0.309–2.448	0.790	0.807	0.264–2.465	0.706
	ER (− vs. +)					0.891	0.208–2.831	0.845	1.379	0.410–4.640	0.604
	PR (− vs. +)					8.187	2.135–31.402	0.002	6.418	1.662–24.785	0.007
	HER2 (+ vs. −)					0.364	0.099–1.346	0.130	0.239	0.055–1.039	0.056
	T (>2–5 cm vs. 0–2 cm)								2.977	0.935–9.480	0.065
	N (N1 vs. N0)								2.506	0.798–7.875	0.116
Positive HER2	*MMP9*	CC + CT vs. TT	0.200	0.104–0.382	0.000	0.143	0.061–0.336	0.000	0.104	0.036–0.301	0.000
	Age (30–40 vs. 41–50 years)		1.898	0.718–5.014	0.196	1.714	0.624–4.707	0.296	1.618	0.563–4.651	0.372
	ER (− vs. +)					1.688	0.515–5.535	0.387	2.090	0.619–7.062	0.235
	PR (− vs. +)					1.254	0.376–4.184	0.712	1.461	0.411–5.187	0.558
	G (G3 vs. G1/G2)								0.276	0.071–1.076	0.064
	T (>2–5 cm vs. 0–2 cm)								1.563	0.513–4.767	0.432
	N (N1 vs. N0)								1.940	0.650–5.790	0.235

OR—odds ratio, CI—confidential interval, vs.—versus, “−“—negative, “+“—positive, ER—estrogen receptor, PR—progesterone receptor, HER2—human epidermal growth factor receptor 2, T—tumor size, N—lymph node involvement (N1—positive, N0—negative), G—tumor histological grade (G1—well differentiated (low grade), G2—moderately differentiated (intermediate grade), G3—poorly differentiated (high grade)).

## Data Availability

The data presented in this study are available on request from the corresponding author.

## Supplementary Materials

The following supporting information can be downloaded at: https://www.mdpi.com/article/10.3390/biomedicines10081891/s1. Table S1. Associations between the polymorphisms and clinicopathological features of breast cancer.
